# Beyond Weight Loss: Comparative Effects of Tirzepatide Plus Low-Energy Ketogenic Versus Low-Calorie Diet on Hepatic Steatosis and Stiffness in MASLD

**DOI:** 10.3390/nu17152409

**Published:** 2025-07-24

**Authors:** Luigi Schiavo, Biagio Santella, Monica Mingo, Gianluca Rossetti, Marcello Orio, Vincenzo Pilone

**Affiliations:** 1 Department of Medicine, Surgery and Dentistry “Scuola Medica Salernitana”, University of Salerno, 84081 Baronissi, Italy; bsantella@unisa.it (B.S.); mmingo@unisa.it (M.M.); 2 National Biodiversity Future Center (NBFC), 90133 Palermo, Italy; 3 General and Bariatric Surgery Unit, Abano Terme Policlinic, 35031 Padova, Italy; gianlucarossetti@yahoo.it; 4 Medical and Diabetological Center (CMSO), 84123 Salerno, Italy; marcello.orio@gmail.com; 5 Public Health Department, University of Naples Federico II, 80131 Naples, Italy; vincenzo.pilone@unina.it

**Keywords:** tirzepatide, MASLD, ketogenic diet, nutritional ketosis, FibroScan, hepatic steatosis, liver stiffness

## Abstract

**Background**: Metabolic dysfunction-associated steatotic liver disease (MASLD) is the most common chronic liver condition globally, strongly linked to obesity, insulin resistance, and type 2 diabetes (T2D). Tirzepatide (TZP), a dual GIP/GLP-1 receptor agonist, improves glycemic control and reduces body weight and the liver fat content in patients with obesity and T2D. However, its effect on liver-specific outcomes such as steatosis and fibrosis remains incompletely characterized. Low-energy ketogenic therapy (LEKT), a nutritional strategy characterized by carbohydrate restriction and nutritional ketosis, may enhance hepatic β-oxidation and reduce hepatic lipogenesis. To date, however, the combination of TZP and LEKT has not been studied in patients with metabolic dysfunction-associated steatotic liver disease (MASLD). This study aimed to compare the hepatic and metabolic effects of TZP combined with either LEKT or a conventional low-calorie diet (LCD) over a 12-week period. **Methods**: Sixty adult patients with MASLD undergoing TZP therapy were prospectively assigned to either an LEKT or a conventional LCD, with 30 participants per group. As primary endpoints, the controlled attenuation parameter (CAP, an index of hepatic steatosis) and liver stiffness measurement (LSM, an index of liver fibrosis) were assessed at the baseline and after 12 weeks using FibroScan^®^. Secondary outcomes included changes in body mass index (BMI), glycated hemoglobin (HbA1c), and liver enzymes. Adherence to both diet and pharmacological treatment, as well as tolerability, were systematically monitored throughout the intervention period. **Results**: Both groups showed significant reductions in body weight (TZP + LEKT, *p* = 0.0289; TZP + LCD, *p* = 0.0278), with no significant intergroup difference (*p* = 0.665). CAP and LSM improved significantly in both groups, but reductions were greater in the TZP + LEKT group (CAP −12.5%, *p* < 0.001; LSM −22.7%, *p* < 0.001) versus LCD (CAP −6.7%, *p* = 0.014; LSM −9.2%, *p* = 0.022). Between-group differences were statistically significant for both CAP (*p* = 0.01) and LSM (*p* = 0.03). **Conclusions**: Based on these preliminary findings, we support the hypothesis that the combination of TZP and LEKT may be superior to TZP with an LCD in reducing hepatic steatosis and stiffness in individuals with obesity.

## 1. Introduction

Metabolic dysfunction-associated steatotic liver disease (MASLD), recently redefined to emphasize its metabolic origins, has become the most prevalent chronic liver disease worldwide, affecting over one-third of the adult population [1]. Its progression from simple steatosis to steatohepatitis, fibrosis, and cirrhosis represents a major public health concern, tightly linked to the global epidemics of obesity, type 2 diabetes mellitus (T2DM), and insulin resistance [2]. Despite its growing burden, no pharmacological agent has yet been formally approved for the treatment of MASLD.

Current clinical guidelines recommend lifestyle modification and weight loss as the cornerstone of treatment, with even modest reductions in body weight (≥7–10%) leading to significant improvements in hepatic histology [3,4]. However, adherence to conventional hypocaloric diets remains suboptimal, and sustainable lifestyle changes are notoriously difficult to implement in real-world settings [5]. Accordingly, recent therapeutic strategies have focused on both pharmacological and nutritional approaches aimed at improving liver-specific outcomes beyond weight loss alone.

Tirzepatide (TZP), a once-weekly dual agonist of the glucose-dependent insulinotropic polypeptide (GIP) and glucagon-like peptide-1 (GLP-1) receptors, has demonstrated remarkable efficacy in inducing weight loss [6], improving glycemic control [7,8], and reducing hepatic fat content [9,10]. Notably, recent phase 2 trials—such as SYNERGY-NASH—have shown that TZP may also promote a histological resolution of steatohepatitis and regression of fibrosis in patients with biopsy-confirmed MASH [10,11]. Nevertheless, the full therapeutic potential of TZP may be further enhanced when combined with metabolically synergistic nutritional interventions [12].

Low-energy ketogenic therapy (LEKT) has attracted increasing interest for its ability to modulate hepatic metabolism through mechanisms distinct from simple caloric restriction [13]. Carbohydrate restriction promotes nutritional ketosis, which enhances hepatic β-oxidation, suppresses de novo lipogenesis, and improves insulin sensitivity [14,15]. Additionally, ketone bodies exert signaling effects that may attenuate hepatic inflammation and fibrogenesis via inhibition of the NLRP3 inflammasome and modulation of PPAR-α and FGF21 activity [16,17].

In parallel, non-invasive imaging techniques such as transient elastography (FibroScan^®^) have become widely accepted for the quantitative assessment of hepatic steatosis and fibrosis, providing practical and reproducible surrogates for histologic endpoints in clinical trials [18,19,20].

Building on this rationale, we recently demonstrated that the combination of TZP and LEKT yields clinically meaningful benefits beyond weight loss alone—most notably, the preservation of fat-free mass, muscle strength, and resting metabolic rate [12]—highlighting the potential of metabolically targeted interventions in the management of MASLD.

Given this background, we hypothesized that combining TZP with an LEKT intervention would result in additive or synergistic effects on hepatic fat and stiffness in patients with MASLD. This study aimed to prospectively evaluate, over a 12-week period, the comparative impact of TZP plus LEKT versus TZP plus a standard low-calorie diet on non-invasively measured hepatic parameters and metabolic outcomes in a cohort of adults with MASLD.

## 2. Materials and Methods

### 2.1. Study Design and Patient Selection

Between October and December 2024, we conducted a prospective, exploratory study involving 60 consecutive adult patients with obesity and a confirmed diagnosis of MASLD who were receiving treatment with tirzepatide (TZP). Eligible participants were aged ≥18 years and had a body mass index (BMI) of ≥30 kg/m^2^, or ≥27 kg/m^2^ in the presence of at least one obesity-related comorbidity, such as type 2 diabetes (T2D), hypertension, dyslipidemia, obstructive sleep apnea, or cardiovascular disease. All participants had previously failed at least one structured dietary intervention aimed at weight reduction [6,21,22,23,24]. The diagnosis of MASLD was confirmed through evidence of hepatic steatosis, as detected by ultrasound with characteristic echogenicity, previous histological confirmation, or CAP values ≥ 248 dB/m obtained via transient elastography [18,19,20]. In accordance with inclusion criteria used in prior TZP trials, exclusion criteria included a history of type 1 diabetes, body weight variability exceeding 5 kg in the prior 90 days, recent or scheduled bariatric procedures, and use of anti-obesity drugs within three months of enrollment [6,21,22,23,24]. Additional exclusion criteria included the presence of decompensated cirrhosis, current or previous malignancies (including hepatocellular carcinoma), active or past alcohol abuse (>20 g/day for women, >30 g/day for men), viral hepatitis, or other chronic liver conditions unrelated to metabolic dysfunction. Also excluded were pregnant individuals, those with diagnosed eating disorders (e.g., bulimia nervosa, binge eating disorder, or night eating syndrome), serum creatinine > 1.8 mg/dL, transaminase levels (GOT or GPT) exceedingly threefold the upper limit of normal, and inability to adhere to dietary protocols for personal, religious, or physical reasons (e.g., chewing or swallowing impairment) [25,26,27].

The present cohort partially overlaps with the one described in a previous publication that evaluated the effects of TZP and LEKT on body composition, muscular function, and resting metabolic rate [12]. In that study, detailed body composition analysis via bioelectrical impedance showed that the TZP + LEKT group experienced significantly greater preservation of fat-free mass and skeletal muscle strength compared to the TZP + LCD group, despite similar reductions in body weight. Given that the current manuscript focuses on hepatic endpoints, these data are not repeated here but are directly relevant to the metabolic profile of the intervention.

However, the current analysis focuses on hepatic endpoints—specifically, changes in liver fat content and stiffness assessed by transient elastography—that were not previously reported. The study complied with the ethical standards of the national and institutional research committees and adhered to the Declaration of Helsinki and its later amendments. Ethical board review was not required, as the study fell under the category of “negligible risk research”, which includes investigations posing no foreseeable harm or discomfort to participants [12]. Tirzepatide, the pharmacological agent used in this study, was not considered investigational, as it had received full approval for the treatment of obesity by both the U.S. Food and Drug Administration (FDA) and the Italian Medicines Agency (AIFA). At the time of the study, only the 2.5 mg and 5 mg doses were commercially available in Italy, although the AIFA had approved six dosage levels ranging from 2.5 mg to 15 mg. Participants were individually counseled before starting TZP therapy and informed about the dietary options to be followed for the 12-week intervention period. Based on feasibility and willingness to comply with the assigned regimen—particularly with the stricter macronutrient composition and monitoring required in LEKT—participants were allocated to one of the two dietary protocols. Allocation was not based on clinical severity, and no significant differences in baseline characteristics (BMI, HbA1c, CAP, LSM) were observed between groups.

Although LEKT and LCD differ substantially in macronutrient composition, they were designed to be isocaloric (~1200 kcal/day) to allow a comparison of their metabolic effects beyond energy restriction. This pragmatic approach was intended to reflect real-world clinical scenarios, in which distinct evidence-based dietary strategies are selected based on patient characteristics, and to evaluate whether combining TZP with a metabolically targeted intervention such as LEKT offers additional hepatic benefits compared to a conventional LCD.

**Figure 1:**
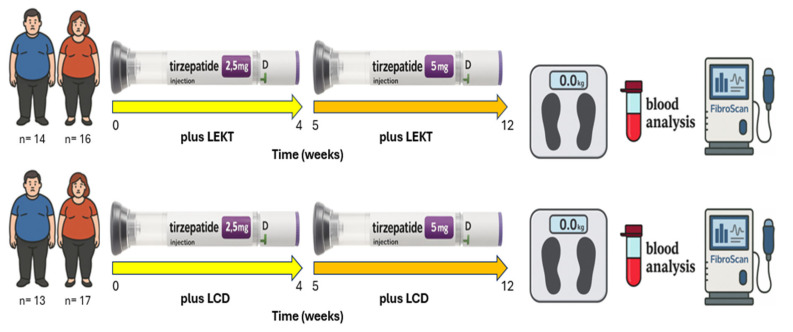
Flow chart comparing a 12-week TZP + LEKT vs. TZP + LCD on total body weight, clinical status, and FibroScan parameters. LEKT = low-energy ketogenic therapy; LCD = low-calorie diet.

Due to the well-recognized role of individual motivation, cognitive readiness, and comprehension in sustaining adherence, we opted for a non-randomized allocation strategy. Patients were assigned to the dietary arm deemed most appropriate for their clinical and behavioral profile (Figure 1). Although randomization remains the gold standard for reducing allocation bias, a pragmatic design was preferred to enhance real-world adherence, as previously emphasized in the literature [12,28,29].

Thus, two groups were formed: one receiving TZP plus LEKT (*n* = 30) and the other TZP plus LCD (*n* = 30). Written informed consent was obtained from all participants following a full explanation of study aims and procedures.

### 2.2. TZP Protocol and LEKT and LCD Characteristics

As illustrated in Figure 1, all participants followed a standardized treatment protocol. Participants began treatment with subcutaneous tirzepatide at 2.5 mg weekly during the initial four weeks, alongside either the LEKT or LCD plan. Subsequently, the dosage was escalated to 5 mg per week for the remaining eight weeks, while maintaining adherence to the assigned nutritional plan. To ensure consistency across the intervention arms, two structured meal plans were designed, one for each dietary group. The LEKT plan was generated using a publicly available ketogenic diet application (https://www.eatthismuch.com), while the LCD plan was formulated using Nutrigeo 8 software (Progeo, Ascoli Piceno, Italy). Both platforms enabled the customization of food quantities based on each participant’s needs.

Both diets were designed to provide approximately 1200 kcal/day. Macronutrient distribution in the LCD plan followed the SURMOUNT-1 model, comprising 50% carbohydrates, 20% proteins, and 30% fats [6]. Conversely, the LEKT plan, as previously described [25,27], provided less than 30 g/day of carbohydrates (~10% of total energy), with 43% of total energy from protein (equivalent to 1.3 g/kg of ideal body weight) and 44% from fat—primarily unsaturated sources. Dietary compliance in the LEKT group was monitored via capillary blood ketone measurements, with >85% of participants achieving β-hydroxybutyrate concentrations ≥ 0.5 mmol/L throughout the intervention period.

Participants were instructed to maintain their habitual physical activity levels throughout the 12-week intervention period, and no additional exercise recommendations were provided. Clinical and metabolic assessments were performed at baseline (one day prior to the initiation of TZP therapy) and at the conclusion of the 12-week follow-up, allowing for the evaluation of treatment effects on liver and metabolic outcomes.

### 2.3. Assessment of BW, CAP, LSM, and Laboratory Parameters

Body weight (BW, in kilograms) and height (in centimeters) were assessed under standardized conditions. Height was measured using a Seca 206 mechanical stadiometer (Intermed, Milano, Italy), and body weight was determined using a Seca 869 flat digital scale (maximum capacity: 250 kg; Intermed, Milano, Italy). All participants underwent vibration-controlled transient elastography (VCTE) using the FibroScan™ 502 Touch system (Echosens, Paris, France), operated by a trained technician. Both M and XL probes were available and selected based on probe-to-liver capsule distance, a measurement used to determine the appropriate probe type. Specifically, the M probe was used when this distance was <25 mm, and the XL probe was selected when the distance exceeded 25 mm. Fibrosis stages were classified using LSM cut-offs: F0–F1 (2–7 kPa), F2 (7–10 kPa), F3 (10–14 kPa), and F4 (≥14 kPa), aligning with standard diagnostic thresholds. Controlled attenuation parameter (CAP) values were expressed in decibels per meter (dB/m) and reported only when the VCTE reading was technically valid, in accordance with device signal quality criteria. CAP values were interpreted as follows: a CAP ≥ 238 dB/m indicated the presence of hepatic steatosis, consistent with a diagnosis of MASLD. Steatosis grading was defined as mild (S1: 238–260 dB/m), moderate (S2: 261–292 dB/m), and severe (S3: ≥293 dB/m). These diagnostic thresholds correspond to manufacturer-provided reference ranges for FibroScan™.

The FibroScan operator was blinded to treatment allocation and was not involved in patient selection, dietary counseling, data analysis, or manuscript preparation.

Standard blood chemistry panels included liver function markers (AST, ALT, and GGT), as well as glucose, insulin, renal function indicators (creatinine, urea, uric acid, BUN), ketone levels, iron status, hemoglobin, and lipid profile parameters such as total cholesterol, HDL, LDL, and triglycerides. All analyses were conducted in an accredited clinical facility, adhering to validated internal procedures and external quality control standards, based on the manufacturer’s standard operating instructions.

### 2.4. Evaluation of Treatment Adherence and Side Effects

Adherence to both dietary protocols and tirzepatide (TZP) treatment was actively monitored throughout the 12-week study period. For the pharmacological intervention, to maintain adherence, tirzepatide was administered exclusively in a hospital setting during scheduled appointments. This protocol guaranteed full compliance and enabled continuous monitoring by healthcare staff, thereby reducing variability related to medication adherence. With respect to diet adherence, participants in the LEKT group were monitored via measurement of capillary blood ketones, which confirmed their maintenance of nutritional ketosis. In addition, changes in body weight were recorded in both groups and used as an indirect marker of adherence to dietary recommendations. These combined strategies supported the overall fidelity and reliability of both the nutritional and pharmacological interventions throughout the trial. To assess potential adverse effects associated with dietary intake, a multimodal, qualitative monitoring approach was implemented. At each monthly dietary counseling session, participants completed a standardized symptom and appetite tracking questionnaire, which gathered data on hunger, satiety, and any adverse gastrointestinal symptoms such as nausea, vomiting, diarrhea, or constipation [6,21,22,23]. All completed forms were independently reviewed by a certified nutritionist, who ensured the accuracy, completeness, and internal consistency of the reported data.

### 2.5. Statistical Analysis

Statistical comparisons between the TZP + LEKT and TZP + LCD groups were carried out for body weight (BW), liver stiffness (LSM), and steatosis (CAP). Within-group changes were assessed using paired *t*-tests, while differences between groups were analyzed using the Mann–Whitney U test. Analyses were performed using GraphPad Prism software (version 9.1.2; GraphPad Software, La Jolla, CA, USA). The choice of statistical method was based on both data distribution and sample size considerations. Data normality was evaluated using the Shapiro–Wilk test, which indicated no significant deviation from normality (*p* > 0.05), justifying the use of parametric tests for within-group evaluations. Nevertheless, due to the relatively small number of subjects (approximately 30 per group) and the presence of slight asymmetry in some variables, non-parametric methods were applied for inter-group comparisons to strengthen result reliability and reduce type I error. This dual-statistical approach allowed for a reliable evaluation of the differential effects of the two interventions on liver-related and metabolic outcomes derived from FibroScan and standard laboratory measures. Results are presented as means ± standard deviation (SD), and statistical significance was defined by a two-sided *p*-value < 0.05. When applicable, p-values below 0.001 were conventionally reported as *p* < 0.001. Percentage changes in BW, LSM, and CAP were calculated and graphically represented to illustrate trends over time. Normal distribution of data was confirmed in both groups (*p* > 0.05) using the Shapiro–Wilk test. Group differences were further examined using independent *t*-tests, where appropriate.

## 3. Results

### 3.1. Characteristics of the Study Groups at Baseline

A total of 60 participants were enrolled in the study, consisting of 33 women and 27 men. Prior to the initiation of TZP therapy, the TZP + LEKT and TZP + LCD groups showed no significant differences in baseline BW, BMI, LSM, or CAP values (Table 1).

### 3.2. Impact of TZP + LEKT vs. TZP + LCD on BW, LSM, and CAP

**Figure 2:**
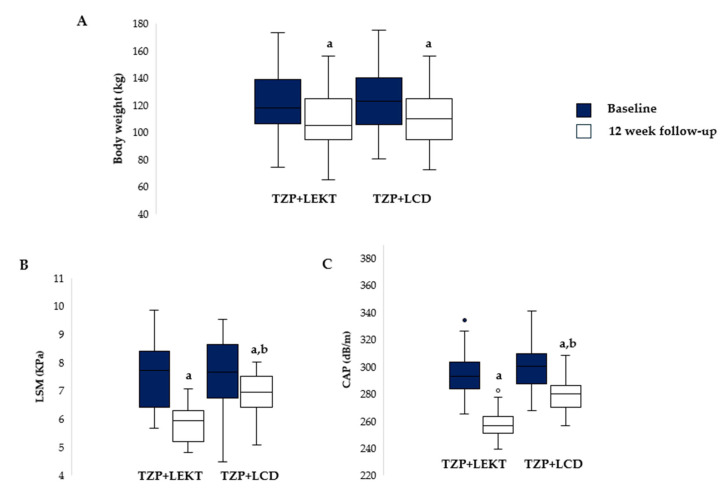
Box plots show baseline and 12-week follow-up changes in body weight (**A**), LSM (**B**), and CAP (**C**) in both groups studied. Horizontal bars represent median values. Lower and upper boundaries of the box represent the first and the third quartiles, respectively. Lower and upper error bars represent the minimum and the maximum values, respectively. TZP = tirzepatide; LEKT = low-energy ketogenic therapy; LCD = low-calorie diet. ^a^ 12-week follow-up vs. baseline; ^b^ 12-week follow-up TZP + LEKT vs. LCD.

All participants completed the study without dropout. After 12 weeks, a significant reduction in body weight was observed in both treatment groups compared to the baseline (TZP + LEKT, *p* = 0.0289; TZP + LCD, *p* = 0.0278). However, the difference in weight loss between the two groups was not statistically significant (*p* = 0.665). The mean percentage reduction in body weight reached −10.2% ± 2.5 in the TZP + LEKT arm and −9.8% ± 2.9 in the TZP + LCD arm (Figure 2A).

Furthermore, as shown in Figure 2B, LSM values significantly decreased in the TZP + LEKT group, from 7.5 ± 1.1 to 5.8 ± 0.6 kPa (*p* < 0.001), indicating a 22.7% reduction in liver stiffness. In the TZP + LCD group, LSM declined from 7.6 ± 1.2 to 6.9 ± 0.7 kPa (*p* = 0.022), corresponding to a 9.2% reduction. Again, the reduction was significantly greater in the LEKT group compared to the LCD group (*p* = 0.03) (Figure 2C).

Similarly, in the TZP + LEKT group, CAP values declined significantly from 295 ± 16 to 258 ± 10 dB/m (*p* < 0.001), reflecting a 12.5% reduction in hepatic steatosis. In the TZP + LCD group, CAP decreased from 298 ± 17 to 278 ± 12 dB/m (*p* = 0.014), corresponding to a 6.7% reduction. The between-group comparison showed a statistically significant greater reduction in CAP in the TZP + LEKT group than in the LCD group (*p* = 0.01). Notably, 26 out of 30 patients (86.7%) in the TZP + LEKT group experienced a reduction in LSM after 12 weeks. In the remaining four patients (13.3%), LSM remained stable or increased marginally (+0.2 to +0.8 kPa), despite weight loss and improved metabolic markers.

### 3.3. Impact of TZP + LEKT vs. TZP + LCD on Clinical Status

Table 2 highlights a marked overall clinical improvement in both treatment groups. Nonetheless, the TZP + LEKT group showed a significantly greater enhancement in both HOMA index and HbA1c levels compared to the TZP + LCD group. As anticipated, follow-up assessments revealed substantially elevated ketone concentrations in participants receiving the LEKT-based intervention.

### 3.4. Effects of TZP + LEKT and TZP + LCD on Appetite Suppression and Tolerability

A higher percentage of individuals in the TZP + LEKT group reported a reduction in appetite compared to those following the TZP + LCD regimen (60% vs. 26.7%), likely reflecting the appetite-suppressing effects of ketogenic nutrition, which is often linked to lower hunger perception. In terms of adverse events, nausea was the most common side effect, affecting 50% of the TZP + LEKT group and 56.7% of the TZP + LCD group. Constipation was also frequently reported, occurring in 53.3% of LEKT participants and 50% of LCD participants. Vomiting was documented in 20% and 23.3% of individuals in the LEKT and LCD groups, respectively. Diarrhea was the least reported symptom, observed in 6.7% of the TZP + LEKT group and in all (100%) of the TZP + LCD group (Table 3).

In general, both nutritional approaches were well accepted by participants, and no serious adverse events were observed. Mild gastrointestinal discomfort occurred at comparable rates in both groups, indicating that these effects were more likely linked to the pharmacological agent than to dietary composition.

## 4. Discussion

This prospective, non-randomized pilot study provides preliminary evidence that the combination of TZP, a dual GIP/GLP-1 receptor agonist, with LEKT leads to significantly greater reductions in hepatic steatosis and stiffness, assessed by FibroScan^®^, compared to TZP combined with a standard LCD in patients with MASLD.

Notably, these hepatic improvements occurred despite comparable weight loss, suggesting that the observed benefits are not solely attributable to energy restriction but rather to distinct metabolic adaptations induced by nutritional ketosis.

**Figure 3:**
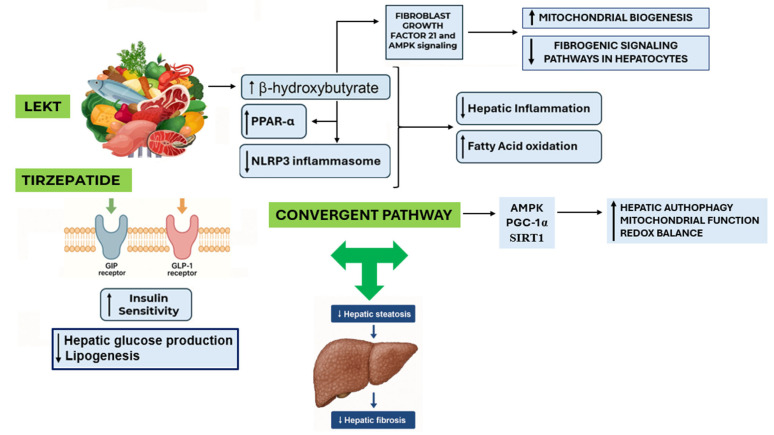
Synergistic mechanisms of tirzepatide and low-energy ketogenic therapy in MASLD. Tirzepatide (TZP) improves insulin sensitivity, and it reduces hepatic glucose production and lipogenesis via dual GIP/GLP-1 receptor agonism. Low-energy ketogenic therapy (LEKT) promotes nutritional ketosis, leading to increased β-hydroxybutyrate levels, PPAR-α activation, and inhibition of the NLRP3 inflammasome, which collectively reduce hepatic inflammation and enhance fatty acid oxidation. Both interventions converge on the AMPK–PGC-1α–SIRT1 axis, resulting in improved hepatic autophagy, mitochondrial function, and redox balance. This shared metabolic convergence leads to reductions in hepatic steatosis and fibrosis, as observed in the clinical outcomes. Legend: (→) indicates stimulation or directional flow; (↑) indicates upregulation or increased activity; (↓) indicates downregulation or suppression; (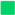
T) indicates convergence of mechanistic pathways shared by TZP and LEKT.

LEKT is known to suppress de novo lipogenesis, enhance hepatic mitochondrial β-oxidation, and modulate insulin and glucagon dynamics [30]. In addition, nutritional ketosis activates peroxisome proliferator-activated receptor alpha (PPAR-α) and increases fibroblast growth factor 21 (FGF21) expression, both of which are central to hepatic lipid catabolism, mitochondrial biogenesis, and metabolic flexibility [31]. Ketone bodies, particularly β-hydroxybutyrate (βHB), act as signaling molecules with anti-inflammatory and antifibrotic properties, notably by inhibiting the NLRP3 inflammasome and NF-κB activity in hepatocytes and Kupffer cells [32]. These actions may collectively attenuate hepatic stellate cell activation and reduce fibrogenesis. TZP’s mechanism of action complements these effects. Its dual incretin agonism improves hepatic insulin sensitivity, suppresses glucagon secretion, enhances glucose disposal, and reduces systemic inflammation [6]. Activation of GIP receptors may also directly modulate hepatic lipid metabolism by increasing adiponectin secretion and improving lipid partitioning [33]. Both agents converge on common metabolic regulators, such as AMP-activated protein kinase (AMPK), PGC-1α, and SIRT1, promoting hepatic autophagy, mitochondrial function, and redox balance [34,35]. The proposed mechanisms underlying this metabolic synergy are illustrated in Figure 3.

The observed reductions in the controlled attenuation parameter (CAP, −12.5%) and liver stiffness measurement (LSM, −22.7%) in the TZP + LEKT group are clinically meaningful. These changes are consistent with histological improvements previously associated with fibrosis regression and steatosis resolution, as reported in studies including the SYNERGY-NASH trial [10]. Although that trial demonstrated TZP’s efficacy in histologically confirmed MASH, the current findings suggest that co-administration with LEKT may enhance or accelerate these effects, even within a 12-week timeframe.

These findings complement previous evidence suggesting that combining TZP with LEKT may offer broader metabolic advantages beyond liver-specific endpoints [12]. In particular, our prior analysis in the same cohort demonstrated a superior preservation of lean body mass and basal metabolic rate in the TZP + LEKT group, reinforcing the concept that nutritional ketosis may help mitigate the catabolic effects of caloric restriction and pharmacotherapy-induced weight loss [12].

Nevertheless, it is important to note that a small subset of patients (13.3%) in the TZP + LEKT group did not exhibit a reduction in LSM, despite achieving substantial weight loss and improvements in glycemic and metabolic parameters. This highlights the existence of interindividual variability in hepatic response, possibly driven by differences in baseline fibrosis stage, inflammatory milieu, or genetic factors. Such variability underscores the need for personalized approaches and longitudinal assessment of liver stiffness dynamics.

Glycemic control, as reflected by HbA1c reductions, and hepatic transaminase improvements (notably ALT) were also more favorable in the TZP + LEKT group, supporting a more pronounced improvement in insulin sensitivity and hepatocellular integrity [10]. These findings are supported by mechanistic data showing that LEKT enhances hepatic AMPK activation and ketogenesis, while tirzepatide increases metabolic efficiency and reduces hepatic glucose production [36].

Both interventions were safe and well tolerated. The incidence of gastrointestinal side effects was mild and similar across groups. No cases of hypoglycemia or pathological ketosis were reported, underscoring the safety of supervised LEKT even when combined with incretin-based pharmacotherapy [12]. Nonetheless, we acknowledge that nutritional ketosis may not be a universally sustainable strategy in the long term. Although the ketonemia levels observed in our study remained within the physiological range, prolonged adherence to LEKT requires close monitoring and individualization. In this light, LEKT + TZP may be best positioned as an intensive, short-to-intermediate-term intervention in patients with advanced metabolic dysfunction, while LCD + TZP may serve as a more suitable long-term maintenance option in selected individuals. Future studies should directly assess these longitudinal trajectories.

The LEKT protocol in this study was intentionally limited to 12 weeks and conducted under clinical supervision with appropriate micronutrient supplementation. Although short-term ketogenic interventions have been shown to be safe and metabolically effective, concerns remain regarding their long-term impact on micronutrient status, acid-base balance, and cardiovascular risk, especially in diets rich in saturated fats. For this reason, the LEKT model employed here prioritized unsaturated fat sources and was not intended as a chronic therapeutic approach. Instead, we propose it as a structured, short-term metabolic intervention that may be followed by a more balanced maintenance diet, tailored to the patient’s long-term needs.

A potential limitation of this study is that the enrolled population consisted predominantly of individuals with morbid obesity (mean BMI > 40 kg/m^2^), which may limit the generalizability to MASLD patients with a lower BMI. However, this phenotype represents a high-risk subgroup with a greater hepatic burden, in whom therapeutic efficacy is particularly relevant. Further studies are warranted to assess whether similar hepatic improvements can be observed in MASLD patients with overweight, normal weight, or lean metabolic phenotypes.

This study presents several notable strengths, including its prospective design, the use of a standardized TZP dosing protocol, objective quantification of hepatic parameters through FibroScan^®^, and real-time monitoring of dietary adherence via blood ketone measurements. Importantly, the trial provides preliminary insights into a novel therapeutic combination that targets both hepatic pathology and systemic metabolic derangements. However, some limitations should be acknowledged. The lack of randomization introduces the possibility of selection bias, as patients were assigned to interventions based on anticipated dietary adherence and individual feasibility. Although this pragmatic, real-world design improves external validity and reflects how treatment decisions are made in clinical practice, it inherently reduces internal validity and prevents definitive causal conclusions.

Additionally, the relatively short follow-up period (12 weeks) precludes the assessment of longer-term effects on histological liver changes, cardiovascular outcomes, and sustainability of metabolic improvements. The modest sample size also limits statistical power and prevents robust subgroup analyses based on fibrosis stage, sex, or baseline metabolic profiles. Nevertheless, given the rising global burden of MASLD and its tight connection with obesity and T2D, there is a critical need for non-invasive, synergistic strategies to improve liver health. Our findings support the potential of combining TZP with metabolically focused nutritional interventions such as LEKT, which may outperform pharmacologic therapy alone, particularly in patients with early-stage disease or those unresponsive to standard dietary approaches. Future randomized controlled trials with larger cohorts, extended follow-up, and histological endpoints are warranted to confirm these observations. Further mechanistic investigations, including hepatic transcriptomics, lipid flux analyses, and integrative metabolomic/proteomic profiling, will be crucial to unravel the molecular synergy between TZP and nutritional ketosis. Emerging targets such as farnesoid X receptor (FXR) signaling and bile acid homeostasis deserve particular attention, as FXR plays a pivotal role in modulating hepatic lipid metabolism, inflammation, and fibrotic remodeling. Similarly, the interplay between mitochondria and peroxisomes in oxidative lipid processing and redox regulation may represent a key axis in the reversal of steatofibrosis, potentially enhanced by both TZP and LEKT. Incorporating multi-omics analyses alongside continuous metabolic monitoring may ultimately enable a more personalized, multimodal approach to MASLD therapy.

## 5. Conclusions

This prospective, non-randomized pilot study provides preliminary evidence that TZP combined with LEKT produces superior hepatic and metabolic outcomes in patients with MASLD compared to TZP paired with an LCD. These benefits appear to extend beyond weight loss, likely reflecting specific and convergent metabolic adaptations. While promising, these findings warrant confirmation in larger, randomized studies before being translated into routine clinical care.

## Figures and Tables

**Table 1:** Characteristics of study groups at baseline. Data are reported as mean ± SD.

	LEKT (*n* = 30)	LCD (*n* = 30)	*p* (LEKT vs. LCD)
Sex (male/female, *n*)	14/16	13/17	-
Body Weight (kg)	121.9 ± 23.6	124.3 ± 23.8	0.70
Body Mass Index (kg/m^2^)	44.9 ± 6.28	45.5 ± 7.35	0.73
LSM (kPa)	7.5 ± 1.1	7.6 ± 1.2	0.81
CAP (dB/m)	295 ± 16	298 ± 17	0.58

LEKT = low-energy ketogenic therapy. LCD = low-calorie diet. LSM = liver stiffness measurement. CAP = controlled attenuation parameter. SD = standard deviation.

**Table 2:** Patients’ clinical parameters at baseline and after 12 weeks. Data are reported as mean ± SD. * statistically significant.

Clinical Parameters (Cut-Off)	Group	Baseline	12-Week Follow-Up	*p*
Glucose	TZP + LEKT	87 ± 21	80 ± 18	0.17
(70–100 mg/dL)	TZP + LCD	92 ± 17	87 ± 13	0.21
Insulin	TZP + LEKT	8.5 ± 6.3	7.1 ± 5.2	0.35
(<25 mU/L)	TZP + LCD	7.7 ± 4.7	7.2 ± 3.1	0.63
HOMA IR Index	TZP + LEKT	1.83 ± 0.8	1.40 ± 1.1	0.09 *
(<2.5)	TZP + LCD	1.75 ± 1.4	1.55 ± 0.96	0.52
Hemoglobin A1C	TZP + LEKT	4.7 ± 1.7	3.7 ± 1.1	0.01 *
(<6.1%)	TZP + LCD	4.3 ± 0.77	4.0 ± 0.80	0.14
Total cholesterol	TZP + LEKT	194 ± 42	162 ± 11	<0.001 *
(<200 mg/dL)	TZP + LCD	176 ± 48	159 ± 23	0.09
HDL	TZP + LEKT	47 ± 12	55.5 ± 18	0.03
(>50 mg/dL)	TZP + LCD	49.9 ± 22	45.4 ± 10	0.31
Triglycerides	TZP + LEKT	151 ± 75	118 ± 28.5	0.03 *
(<150 mg/dL)	TZP + LCD	116 ± 54	109 ± 18.2	0.50
Creatine	TZP + LEKT	0.76 ± 0.19	0.81 ± 0.26	0.40
(0.55–1.02 mg/dL)	TZP + LCD	0.79 ± 0.17	0.83 ± 0.15	0.34
AST	TZP + LEKT	23 ± 13.8	24 ± 8.2	0.73
(<34 U/L)	TZP + LCD	26 ± 22.9	29 ± 9.5	0.51
ALT	TZP + LEKT	29 ± 21.1	19 ± 3.7	0.01
(<55 U/L)	TZP + LCD	35 ± 32.4	29 ± 8.7	0.33
GGT	TZP + LEKT	32 ± 26.4	28 ± 6.9	0.42
(<38 U/L)	TZP + LCD	31 ± 21.1	29 ± 9.2	0.64
Ketonemia	TZP + LEKT	0.3 ± 0.04	0.82 ± 0.49	<0.001
(<0.6 mmol/L)	TZP + LCD	0.25 ± 0.07	0.27 ± 0.05	0.21

LEKT = low-energy ketogenic therapy. LCD = low-calorie diet. HOMA IR Index = Homeostatic Model Assessment of Insulin Resistance Index. HDL = high-density lipoprotein. AST = aspartate aminotransferase. ALT = alanine aminotransferase. GGT = gamma-glutamil transferase. SD = standard deviation.

**Table 3:** Patient-reported frequencies of reduced appetite and gastrointestinal symptoms (nausea, vomiting, constipation, and diarrhea) observed during the 12-week course of TZP treatment in the LEKT and LCD groups.

Patient-Reported Outcomes	LEKT (*n* = 30)	LCD (*n* = 30)
Appetite reduction (%)	18	8
Nausea episodes (%)	15	17
Vomiting episodes (%)	6	7
Constipation episodes (%)	16	15
Diarrhea episodes (%)	4	3

LEKT = low-energy ketogenic therapy. LCD = low-calorie diet.

## Data Availability

The data included in this manuscript were derived from the University database. We are not authorized to share the data with third-party organizations. However, the corresponding author is available to provide any explanation to the Editor if requested.
